# Integrated omic analysis provides insights into the molecular regulation of stress tolerance by partial root-zone drying in rice

**DOI:** 10.3389/fpls.2023.1156514

**Published:** 2023-06-09

**Authors:** Minhua Zhao, Canghao Du, Jian Zeng, Zhihong Gao, Yongyong Zhu, Jinfei Wang, Yupeng Zhang, Zetao Zhu, Yaqiong Wang, Mingjie Chen, Yuesheng Wang, Junli Chang, Guangxiao Yang, Guangyuan He, Yin Li, Xiaoyuan Chen

**Affiliations:** ^1^ Henry Fok School of Biology and Agriculture, Guangdong Engineering Technology Research Center for Efficient Utilization of Water and Soil Resources in North Region, Shaoguan University, Shaoguan, Guangdong, China; ^2^ The Genetic Engineering International Cooperation Base of Chinese Ministry of Science and Technology, Key Laboratory of Molecular Biophysics of Chinese Ministry of Education, College of Life Science and Technology, Huazhong University of Science and Technology, Wuhan, Hubei, China

**Keywords:** RNA-Seq, metabolomics, omics analysis, rice, osmotic stress, regulation of gene expression, transcription factors, partial root-zone drying (PRD)

## Abstract

Partial root-zone drying (PRD) is an effective water-saving irrigation strategy that improves stress tolerance and facilitates efficient water use in several crops. It has long been considered that abscisic acid (ABA)-dependent drought resistance may be involved during partial root-zone drying. However, the molecular mechanisms underlying PRD-mediated stress tolerance remain unclear. It’s hypothesized that other mechanisms might contribute to PRD-mediated drought tolerance. Here, rice seedlings were used as a research model and the complex transcriptomic and metabolic reprogramming processes were revealed during PRD, with several key genes involved in osmotic stress tolerance identified by using a combination of physiological, transcriptome, and metabolome analyses. Our results demonstrated that PRD induces transcriptomic alteration mainly in the roots but not in the leaves and adjusts several amino-acid and phytohormone metabolic pathways to maintain the balance between growth and stress response compared to the polyethylene glycol (PEG)-treated roots. Integrated analysis of the transcriptome and metabolome associated the co-expression modules with PRD-induced metabolic reprogramming. Several genes encoding the key transcription factors (TFs) were identified in these co-expression modules, highlighting several key TFs, including TCP19, WRI1a, ABF1, ABF2, DERF1, and TZF7, involved in nitrogen metabolism, lipid metabolism, ABA signaling, ethylene signaling, and stress regulation. Thus, our work presents the first evidence that molecular mechanisms other than ABA-mediated drought resistance are involved in PRD-mediated stress tolerance. Overall, our results provide new insights into PRD-mediated osmotic stress tolerance, clarify the molecular regulation induced by PRD, and identify genes useful for further improving water-use efficiency and/or stress tolerance in rice.

## Introduction

The adverse impacts of abiotic stresses (such as drought, high salinity, cold, and high light intensity stress) on crop growth and production have becoming greater than before, threatening global food security (Suzuki et al., 2014). Developing new varieties with improved abiotic stress resistance and applying new cultivation techniques are important approaches to attenuate effects of stress on crop production. Recent climate changes have led to globally imbalanced rainfall and droughts (FAO, 2019). Drought stress has become particularly impactful in global agriculture, causing a loss of ~$30 billion in crop production (Gupta et al., 2019). In addition, agricultural activities will require more water to feed the world’s population, which is predicted to reach ~10 billion by 2050 (United Nations, 2011; Koncagül et al., 2018).

The partial root-zone drying (PRD) technique represents a water-saving irrigation strategy to sustain crop growth with limited water resources but without significant yield loss (Mehrabi and Sepaskhah, 2019). The PRD approach has been successful for several major crops, including wheat, rice, maize, potato, and tomato (Kang et al., 2000; Saeed et al., 2008; Ahmadi et al., 2010; Sepaskhah and Ahmadi, 2012; Casa and Rouphael, 2014; Gao et al., 2021). In principle, the PRD technique splits the root system into two parts: one part of the root system in dry soil responds to drought stress and induces related signal transduction, whereas the other part of the root system maintains a normal water status to sustain plant growth (Khalil and Grace, 1993).

When the root system senses drought conditions, hydraulic and abscisic acid (ABA)-related signals are sent, both of which can mediate long-distance root-to-shoot communication to trigger a series of changes at the physiological, metabolic, and gene expression levels (Zhang et al., 2012; Gorgues et al., 2022). The small peptide CLAVATA3/EMBRYO-SURROUNDING REGIONRELATED 25 (CLE25) is synthesized and transported to the leaf tissue to augment ABA biosynthesis and signaling and to induce the expression of downstream stress-responsive genes (Sah et al., 2016; Takahashi et al., 2018). ABA signaling also leads to stomatal closure, which reduces water transpiration (Chen et al., 2020). Water scarcity also causes secondary stresses in plant tissues, such as osmotic and oxidative stresses (Zhang et al., 2012). It has been reported that PRD works for crops in which stomatal movements are sensitive to ABA (Dbara et al., 2016) and that PRD can decrease water use by 50% in grapevines (Collins et al., 2010).

Although PRD is useful for improving stress tolerance and limiting the use of water resources, the underlying mechanisms remain elusive. Previous physiological studies employed PRD experiments to investigate the response of rice seedlings to mild and severe drought treatments and demonstrated the involvement of both ABA and hydraulic signals in this process (Siopongco et al., 2008; Siopongco et al., 2009). However, we hypothesized that PRD-mediated stress tolerance might involve complex stress regulatory networks other than the ABA-dependent pathway. Several important questions regarding PRD-mediated stress tolerance remain unanswered: (1) what major changes occur in drying roots and well-watered roots, respectively, at the molecular level; (2) by which mechanisms does PRD mediate water and nutrient resource utilization to maintain growth with reduced water availability; (3) what are the important genes involved in this process, and can these genes be utilized in genetic improvement for better stress tolerance or water-use efficiency?

In the present study, rice seedlings were used as a research model to obtain evidence supporting our hypothesis and gain insights into PRD-mediated stress tolerance. Rice (*Oryza sativa* L.) was chosen for several reasons. (1) Rice is one of the most important crops globally, and Asia is a prominent producer and consumer of rice (Muthayya et al., 2014); (2) Except for the upland cultivars, rice plants are usually grown in paddy fields and have shallow root systems, demanding a large amount of water to maintain growth (Samson et al., 2002; Yu et al., 2020); (3) When drought occurs, the root system of rice is exposed to anaerobic flooding and aerobic drought conditions alternately, somewhat mimicking the PRD experiment. In this study, we combined physiological measurements, transcriptomics, and metabolomics to characterize the early response of rice seedlings to polyethylene glycol (PEG)-induced stress and PRD-mediated stress tolerance. Integrated omics analysis and our customized transcription factor (TF)-centric method helped to identify several key TFs and target genes involved in metabolic reprogramming during stress- or PRD-mediated tolerance. This provided important candidate genes that warrant functional studies on their roles in the regulation and tolerance of osmotic stress.

## Materials and methods

### Experimental design

**Figure 1:**
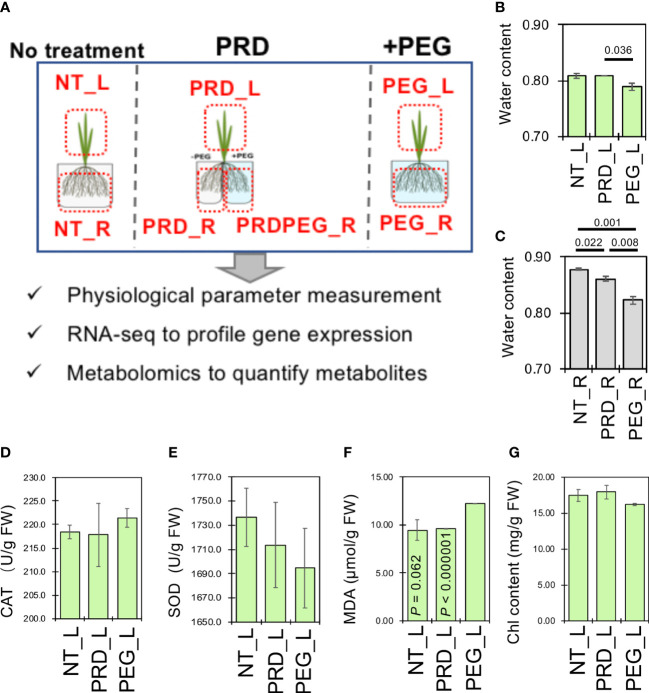
Partial root-zone drying attenuates the osmotic stress-induced water loss in the root. **(A)** Schematic diagram showing the experimental design to reveal the mechanism of enhanced osmotic-stress tolerance caused by PRD. The relative water contents of the leaf **(B)** and root samples **(C)**, the content of catalase (CAT, **(D)**, superoxide dismutase (SOD, **(E)**, malonic dialdehyde (MDA, **(F)** and chlorophyll **(G)** were measured to reflect the physiological status of the leaf tissues. All measurements were performed in triplicates. Statistical difference was determined by pair-wise comparison between the leaf or root samples by using Student’s *t*-test (*P*< 0.05).

In the present study, “MeiXiangZhan No.2”, a representative elite fragrant rice variety widely cultivated in South China, was used (Mo et al., 2017). After seeds germination, the seedlings were cultivated at 32°C (12 h day and 12 h night) in the growth chamber until the four-leaf stage. To investigate the molecular mechanisms involved in PRD-mediated stress tolerance, a hydroponic experimental system was established to partially mimic the osmotic conditions of drought stress and PRD (**Figure 1A**). A set of three experiments was performed in triplicate: (1) the non-treated control experiment (abbreviated as “NT”): the rice seedlings at the four-leaf stage were hydroponically cultivated in growth tubes containing Hoagland nutrient solution (pH 5.5) that was changed every two days; (2) the PRD experiment: the rice seedlings were hydroponically cultivated with the roots separated into two growth tubes; half of the roots were grown in the Hoagland nutrient solution (abbreviated as the PRD treatment) and the other half were grown in PEG solution (100 g/L (m/v) PEG-6000) (abbreviated as the PRDPEG treatment); (3) the PEG treatment experiment: the rice seedlings were hydroponically cultivated in the same PEG solution as in he PRDPEG treatment. In the experiment, PEG, a widely used non-ionic, nonpenetrating high-molecular-weight molecule to induce osmotic stress of root tissues was chosen to mimic the water deficit conditions which rice plants under drought stress in the field may be experienced (Agrawal et al., 2016; Ogbaga et al., 2020). All the rice seedlings were cultivated in three replicates in a growth chamber of 24°C and a photoperiod of 14/10 h of day/night. To minimize potential differences between individual plants, each replicate comprised 12 rice plants that were randomly placed among replicates to remove positional effects on plant growth in the chamber. All plants were treated for six days, and then the mature leaves and roots were sampled, snap-frozen in liquid nitrogen, and stored at −80°C. Within each replicate, the leaf or root tissues were collected from 12 plants and pooled to form three samples (for the leaf and root tissues, respectively) with each sample used for physiological parameter measurements, RNA-seq, and metabolome analysis, respectively.

### Measurement of physiological parameters

Relative water content (RWC) was measured according to Wei et al. (2014). The dehydrated leaves were soaked in distilled water for 4 h, and the turgid weight was recorded. The leaves were dried at 80°C for 48 h to measure the total dry weight. RWC was calculated as follows: RWC = (desiccated weight − DW)/(TW − DW). Chlorophyll content was determined using a UV spectrophotometric method (Feng et al., 2019). The malondialdehyde (MDA), catalase (CAT), and superoxide dismutase (SOD) levels were measured using assay kits (Nanjing Jiancheng Bioengineering Institute, Nanjing, China) (Qiu et al., 2021).

### Transcriptome analysis

Total RNA was extracted using TRIzol reagent. The quality of the extracted RNA samples was examined using agarose gel electrophoresis, a NanoDrop 2000, and an Agilent 2100 Bioanalyzer. Standard protocols for the BGI genomic DNBSEQ-T7 platform were used to construct rice mRNA libraries. RNA-seq libraries were sequenced to generate 150-bp pair-end reads. For sequence quality control, Cutadapt and the FASTX-Toolkit (http://hannonlab.cshl.edu/fastx_toolkit/) were used to trim low-quality base pairs, as previously described (Li et al., 2019a). Clean quality-filtered reads were mapped to the rice reference genome (*var*. Nipponbare IRGSP v1.0) using HISAT2 v2.0.1-beta with default parameters (Kawahara et al., 2013). Only uniquely-mapped reads were retained, and the read count matrix was subjected to differential expression analysis with DEseq2 using the following criteria: fold change ≥ 2 and a false discovery rate (FDR)-adjusted P-value < 0.05. The fragments per kilobase of exons per million mapped sequence reads (FPKM) were calculated for each gene model using RSEM (Li and Dewey, 2011). Genes with at least five mapped reads and an average FPKM ≥ 1 for the three replicates were considered to be expressed. The RNA statistics are provided in the supplementary file (**Supplementary Table S7**).

### Quasi-targeted metabolome analysis

Quasi-targeted metabolomic analysis was performed to identify and quantify metabolites in rice samples, according to previous studies with modifications (Ali et al., 2008; Mark et al., 2010; Yang et al., 2014). The details of this process are described below.

#### Metabolites extraction

Tissues (100 mg) were individually grounded in liquid nitrogen, and the homogenate was resuspended in 500 μL prechilled 80% methanol by vortexing. The samples were incubated on ice for 5 min and centrifuged at 15,000 × *g* and 4°C for 20 min. A fraction of the supernatant was diluted to a final concentration containing 53% methanol with LC-MS grade water. Then, the samples were transferred to a fresh Eppendorf tube and then were centrifuged at 15000 × *g* and 4°C for 20 min. Finally, the supernatant was analyzed using an LC-MS/MS system analysis (Zhou et al., 2021; Peng et al., 2022).

#### HPLC-MS/MS analysis

The LC-MS/MS analyses were performed using an ExionLC AD system (SCIEX, Framingham, MA, USA) coupled with a QTRAP 6500+ mass spectrometer (SCIEX). Samples were injected onto an Xselect HSS T3 (2.1×150 mm, 2.5 μm) column and eluted using a 20-min linear gradient at a flow rate of 0.4 mL/min for the positive/negative polarity mode. Eluent A was 0.1% formic acid-water, while eluent B was 0.1% formic acid-acetonitrile (Ali et al., 2008). The solvent gradient was set as follows: 2% B, 2 min; 2–100% B, 15.0 min; 100% B, 17.0 min; 100–2% B, 17.1 min; 2% B, 20 min. The QTRAP 6500+ mass spectrometer was operated in positive polarity mode with curtain gas of 35 psi, collision gas of medium, ion spray voltage of 5500 V, temperature of 550 °C, ion source gas of 1:60, and ion source gas of 2:60. QTRAP 6500+ mass spectrometer was operated in negative polarity mode with the following parameters: curtain gas of 35 psi, collision gas of medium, ion-spray voltage of −4500 V, temperature of 550 °C, ion source gas of 1:60, ion source gas of 2:60.

#### Metabolites identification and quantification

The detection of experimental samples using multiple reaction monitoring (MRM) was based on an in-house database (Zhang et al., 2022a). More than 3200 commercially available purified standard compounds were registered on an LC-MS/MS platform in the in-house library of Novogene Co., Ltd. (Beijing, China) to determine their characteristics. Retention time (RT) with a narrow RT window, accurate mass match to the library entries (+/− 0.005 amu), Q1 (parent ion), Q3, and the MS/MS forward and reverse scores between the experimental data and authentic standards were applied as criteria to accurately identify biochemicals (Want et al., 2012; Sellick et al., 2011). The data files generated using HPLC-MS/MS were processed using SCIEX OS (v1.4) to integrate and correct the peaks with the following parameter settings: minimum peak height, 500; signal/noise ratio, 5; Gaussian smoothing width, 1. The area of each peak represents the relative contents of the corresponding substances.

#### Metabolomic data analysis

The identified metabolites were annotated using the Kyoto Encyclopedia of Genes and Genomes (KEGG) database (Luo et al., 2015), the Human Metabolome Database (HMDB) database (Yuan et al., 2012) and the Lipidmaps database (Barri and Dragsted, 2013). Principal component analysis (PCA) and partial least squares discriminant analysis (PLS-DA) were performed using metaX63. We applied a univariate analysis (*t*-test) to calculate statistical significance (*P*-value). The metabolites with variable important in projection value VIP > 1 and *P*-value < 0.05 and log2 (fold change) ≥ 1 or ≤ -1 were considered to be differential metabolites. Z-scores were calculated for each differential metabolite to obtain its relative abundance (**Supplementary Table S5**) and were used for k-means clustering analysis to reveal the representative trend within each group of differentially expressed metabolites (DEMs). KEGG enrichment analysis was performed for the DEMs using a relatively loose threshold (*P* < 0.1), as the metabolome data tended to capture a small fraction of all metabolites in the tissue.

### Integrated analysis of transcriptome and metabolome data

Differentially expressed genes (DEGs) obtained from the RNA-seq analysis were subjected to K-means clustering to identify co-expression groups (also known as modules) in which genes are expressed in a similar trend. Many of them tend to share similar functions, are located in the same/related pathways, or have regulatory relationships. K-means clustering analysis, rather than weighted co-expression network analysis (WGCNA), was selected because the limited sample number may result in an unsatisfactory performance for WGCNA (Langfelder and Horvath, 2008; Langfelder et al., 2011). Figure of merit analysis was performed prior to k-means clustering to determine the appropriate number of clusters and ensure the performance of the k-means analysis (**Supplementary Figure S1**) (Yeung et al., 2001). Pearson’s correlation analysis was used to link the co-expression modules and corresponding metabolic clusters.

Enrichment analysis was performed for each module using Gene Ontology (GO) annotation with ClusterProfiler (**Supplementary Table S3**) to obtain functional insights into the co-expression modules (**Supplementary Table S3**) (Wu et al., 2021). The MapMan annotation of the rice genes was downloaded and used to understand the functions of the genes regulated by TFs and regulators (Usadel et al., 2009). Annotated transcription factors in the rice genome were obtained from planTFDB (Jin et al., 2014).

To perform TF-centric analysis, planTFDB-annotated TFs within the co-expression modules were searched using the FunRicegenes database to obtain related functional studies (Yao et al., 2018; Huang et al., 2022). Only TFs or regulators (e.g., TZF7) studied using transgenic or mutant lines were chosen for further analysis. The TFs were separated into two types: (1) representative target genes available: TFs without genome-wide expression analysis data or expression data not available, and the studies of these TFs included functional evidence of the downstream target genes; (2) genome-wide target genes available: TFs with genome-wide expression analysis data available to retrieve the TF’s target genes or regulated genes. For the second type, the genome-wide target genes of a certain TF were retrieved from the studies (**Supplementary Table S7**) to compare whether the target genes were enriched in a particular co-expression module by using a hypergeometric test with R (Li et al., 2019a; Tu and Li, 2020). When the significantly enriched (*P_hypergeometric_* < 0.01) target genes and the corresponding TF were within the same module or meta-module, we presumed that the TF likely regulates the target genes.

### Statistic analysis

Statistical analysis of the physiological parameters was performed with R using *Student*’s t-test (*P* < 0.05).

## Results and discussion

### Physiological changes during the partial root-zone drying and osmotic stress treatments

When osmotic stress occurs, roots have difficulty absorbing water to sustain growth, which in turn triggers hydraulic signals and other chemical and phytohormone signals (e.g., the ABA signal) to the ground tissues to reduce water loss. Osmotic stress usually decreases relative water content and dry weight (Rahman et al., 2023). The relative water content was measured in the leaf and root tissues to examine the effects of PEG- and PRD-treatments (**Figures 1A–C**). Interestingly, significant water loss in the leaves was only induced in the PEG treatment (~0.79) and not in the PRD treatment (~0.81; **Figure 1B**). In contrast, PRD-treated roots had an intermediate water content (~0.86), which was significantly lower than that of the non-treated roots (0.88), but higher than that of the PEG-treated roots (0.82; **Figure 1C**), suggesting that the PRD strategy indeed mitigated osmotic stress or led to osmotic stress tolerance.

Osmotic stress concomitantly disrupts antioxidant defenses due to the higher production of reactive oxygen species (ROS). It could lead to membrane lipid peroxidation indicated by the high amount of MDA and activities of antioxidant enzymes such as SOD and CAT significantly regulated. Furthermore, levels of oxidative stress markers were measured to support this conclusion. In the leaf tissues, non-treated and PRD-treated leaves (NT_L and PRD_L, respectively) appeared to have relatively higher superoxide dismutase enzyme activity (albeit not significantly) but lower catalase enzyme activity when compared to PEG-treated leaves (**Figures 1D, E**). Untreated and PRD-treated leaves also showed lower MDA content than the PEG-treated leaves, indicating less membrane lipid damage (**Figure 1F**).

Moreover, osmotic stress signals have been reported to regulate photosynthesis, carbohydrate production, and energy metabolism (Shu et al., 2010). The chlorophyll contents of the untreated and PRD-treated leaves were higher than that of the PEG-treated leaves, indicating PRD-mediated stable photosynthesis (**Figure 1G**). Overall, the PRD treatment attenuated PEG-induced osmotic stress in plants: the PRD-treated roots were somewhat affected by the osmotic stress while the PRD-treated leaves may have a similar physiological status compared to that of the control leaves, reflected by the results of relative water content, and chlorophyll and MDA contents.

### Transcriptome analysis

**Figure 2:**
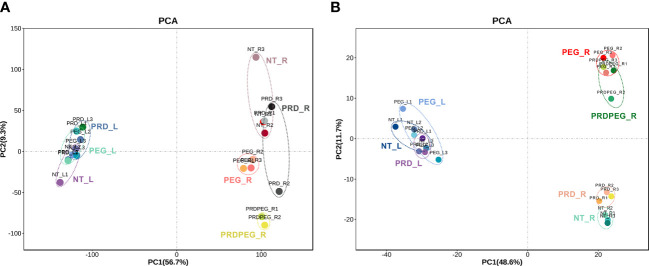
Principal component analysis (PCA) results for the transcriptomic data **(A)** and metabolomic data **(B)**. The abbreviations for the samples used hereafter: NT_L, non-treatment leaf samples; PRD_L, the leaf samples treated by PRD; PEG_L, the leaf samples treated by PEG; NT_R, the nontreatment root samples; PEG_R, the root samples treated by PEG; PRD_R, the root samples from the PRD treatment without PEG-induced osmotic stress; PRDPEG_R, the root samples from the PRD treatment with PEG-induced osmotic stress.

To obtain molecular insights into the PRD-mediated stress tolerance, replicates of seven samples (NT_L, NT_R, PRD_L, PRD_R, PRDPEG_R, PEG_L, and PEG_R; suffix L and R indicating leaves and roots, respectively) were subjected to transcriptome and metabolome analyses. RNA-seq identified approximately 17000 and 21000 genes expressed in the leaf and root samples, respectively (the genes with an average FPKM >= 1 were considered as expressed as described in the methods section. **Supplementary Table S1**). Differential expression analyses of the leaf and root samples led to 6459 differentially expressed genes (DEGs)(the expression matrix is provided in **Supplementary Table S2**). PCA revealed the following: (1) the leaf samples without treatment or those treated with PRD or PEG could not be well separated; (2) in the PRD experiment, the non-treated root (PRD_R) samples were grouped with the control roots (NT_R), whereas the PRD-treated root (PRDPEG_R) samples were clustered with the PEG-treated roots (PEG_R) (**Figure 2A**). This suggests that in the PRD treatment, the roots not experiencing osmotic stress had transcriptomes similar to that of the control plants.

**Figure 3:**
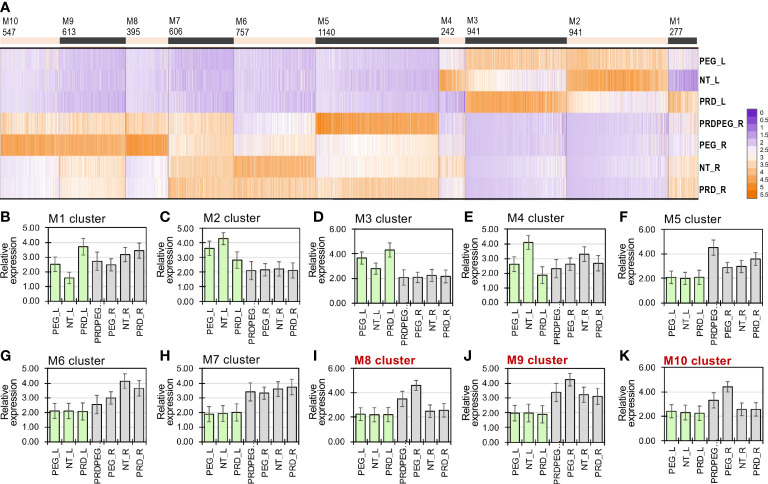
Transcriptomic analysis revealing major differences between the root samples subjected to the osmotic stress (PEG) and partial root-zone drying (PRD) **(A)**. The representative expression patterns of each module are shown in Figures **(B–K)**. The co-expression modules M8, M9 and M10 differed between PRD_R and PRDPEG_R are highlighted in red.

To further dissect the group of genes that may explain the transcriptomic differences induced by PEG or PRD treatment, DEGs were clustered by the k-means approach, yielding ten co-expression modules (i.e., M1 to M10 in **Figure 3A**; **Supplementary Figure S1**), with each of the representative expression patterns shown in **Figure 3**. In the gene modules M2, M3, and M7, the gene expression between the root samples was not significantly different, whereas a much larger number of DEGs (i.e., 4058 genes from the M5 to M10 modules) did not show differential expression among the leaf samples, consistent with the PCA results that the PRD or PEG treatments influenced more genes in the roots than in the leaves. In the modules where genes were differentially expressed among the leaf samples (i.e., M1, M2, M3, and M4), the genes exhibited similar expression trends in PEG_L and PRD_L, which were upregulated or downregulated simultaneously (**Figures 3B–E**). In contrast, the genes in modules M8, M9, and M10 showed the highest expression in PEG_R, intermediate expression in PRDPEG_R, and lowest expression in both NT_R and PRD_R. Particularly, M5 had high expression exclusively in PRDPEG_R, suggesting that this group of genes represents the unique transcriptomic feature of PRDPEG_R, possibly explaining adjustments or communications between the root zones from the untreated and PRDPEG-treated parts. Furthermore, these modules were grouped into three meta-modules (MMA, MMB, and MMC) by calculating the correlations between the module expression patterns (**Supplementary Figure S2**). The MMA generally included three modules: M1, M2, and M4, in which M2 and M4 were positively correlated but negatively associated with M1. MMB included M5, M6, and M7, whereas MMC included M8, M9, and M10. In the MMA, the genes were mainly differentially expressed between leaf samples, possibly explaining the transcriptomic differences between NT_L and PEG_L/PRDPEG_L (**Figures 1B–D**).

### Metabolome analysis

**Figure 4:**
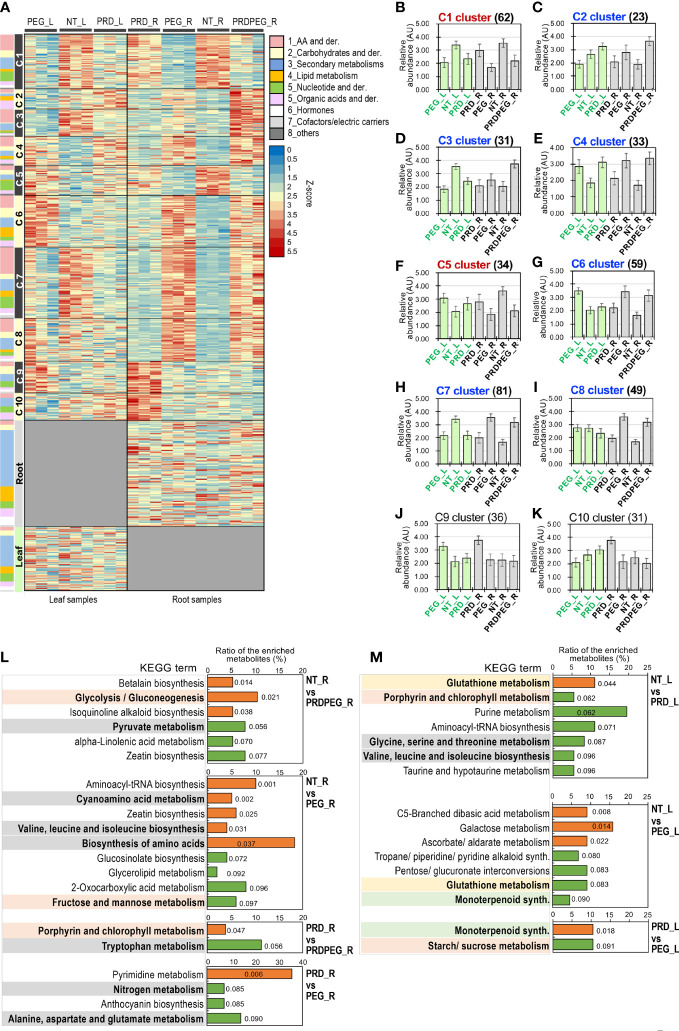
Metabolomic analysis revealing major differences between the root samples subjected to the osmotic stress (PEG) and PRD. **(A)** The heatmap of differential metabolites and those specifically detected in leaves or roots, with the classification of the metabolites indicated using color-codes as shown in the illustration. K-means clustering identified a total of ten metabolic clusters, namely C1, C2, C3, C4, C5, C6, C7, C8, C9 and C10. **(B)** The metabolic dynamic of the cluster C1. **(C)** The metabolic dynamic of the cluster C2. **(D)** The metabolic dynamic of the cluster C3. **(E)** The metabolic dynamic of the cluster C4. **(F)** The metabolic dynamic of the cluster C5. **(G)** The metabolic dynamic of the cluster C6. **(H)** The metabolic dynamic of the cluster C7. **(I)** The metabolic dynamic of the cluster C8. **(J)** The metabolic dynamic of the cluster C9. **(K)** The metabolic dynamic of the cluster C10. The number of metabolites for each cluster are indicated in the bracket. The clusters indicating high metabolic abundance for the NT_R and PRD_R samples but low abundance for the PEG_R samples are written in red, while the clusters indicating low metabolic abundance for the NT_R and PRD_R samples but high abundance for the PEG_R samples are written in blue. **(L, M)** KEGG enrichment analysis of the differential metabolites reflects differences in primary and secondary metabolism between the control, PEG-treated and PRD-treated samples. Comparison of the enriched KEGG metabolic pathways in the roots and leaves are shown in **Figures 4A, B**, respectively. The metabolic pathways related to amino acids/nitrogen, carbohydrates or secondary metabolism are highlighted in grey, light red and green backgrounds, respectively. P values for the enriched KEGG terms are indicated by colors (orange indicating *P*<0.05, and green indicating *P*> 0.05 and <0.1).

To gain a more comprehensive insight into metabolism during PEG and PRD treatments, quasi-targeted metabolomics was employed to identify and profile the metabolites (Zhang et al, 2022a). In the leaf samples, 1230 metabolites were identified, of which 1184 were reproducibly quantified (CV between replicates < 0.8) (**Supplementary Table S4**). In the root samples, 1057 metabolites were identified, with 944 metabolites reproducibly quantified (**Supplementary Table S5**). Pairwise comparison of the metabolite quantities within the leaf and root samples identified 216 DEMs in the leaf and 508 DEMs in the root, yielding a total of 633 DEMs, 73 leaf-specific metabolites, and 121 root-specific metabolites (**Figure 4A**; **Supplementary Table S6**). The larger number of DEMs in the roots than in the leaves further supports the findings based on our physiological and transcriptomic data: the leaf samples had a relatively more stable physiological status than the root samples. PCA result showed that: (1) PC1 and PC2 corresponded to the differences between tissues and treatments, respectively; (2) the metabolic status of the leaf samples (i.e., NT_L, PEG_L, and PRD_L) were similar between each other, while the root metabolic profiles were differentiated probably by the treatments. According to the PCA results, the PRD_R samples appeared to have an intermediate metabolic status between those of NT_R and PEG_R, with PRDPEG_R showing a distinct metabolic pattern (**Figure 2B**). A similar pattern between transcriptome- and metabolome-based PCAs emphasizes that the root, not the leaf, is the main tissue where reprogramming, either induced by PEG or PRD, occurs at the transcriptomic and metabolic levels.

K-means clustering enabled the DEMs falling into ten metabolic clusters (C1 to C10), as well as one cluster for leaf-specific metabolites and one cluster for root-specific metabolites (**Figure 4A**). The representative dynamics of each cluster is shown in **Figures 4B–K**. Because metabolic differences in the root may largely account for PRD-mediated stress tolerance, we highlight two types of metabolic dynamics: (1) the metabolites are highly abundant in NT_R and PRD_R, but in low abundance in PRDPEG_R and PEG_R (i.e., C1 and C5); (2) these metabolites are present at high levels in PRDPEG_R and PEG_R but at low levels in NT_R and PRD_R (i.e., C2, C3, C4, C6, C7, and C8). Also, the metabolites that differed for the comparisons “NT_R vs. PEG_R,” “NT_R *vs* PRDPEG_R,” or those that differed when comparing PRD_R with PRDPEG_R or with PEG_R drawn our attentions. KEGG enrichment analysis for these sets of DEMs identified several metabolic terms associated with primary metabolisms, such as glycolysis/gluconeogenesis, pyruvate metabolism, fructose and mannose metabolism, and chlorophyll metabolism (**Figure 4L**). In particular, the KEGG enrichment results demonstrated that the differences between NT_R vs. PEG_R/PRDPEG_R and PRD_R vs. PEG_R/PRDPEG_R lie in nitrogen and amino acid metabolic pathways, such as valine, leucine, and isoleucine biosynthesis, and alanine, aspartate, and glutamate metabolism. In contrast, the metabolic differences between NT_L vs. PEG_L/PRD_L reside not only in primary metabolic pathways (e.g., chlorophyll and amino acid metabolism) but also in secondary metabolism (monoterpenoid synthesis) and pathways associated with ROS scavenging (glutathione metabolism) (**Figure 4M**). Therefore, our analyses revealed metabolic reprogramming in PEG-induced roots and, more importantly, in PRD-treated roots, suggesting a role for such reprogramming in PRD-mediated osmotic stress tolerance.

### Integrated omics analysis highlights metabolic reprogramming in the PRD-treated roots and identifies important transcription factors and candidate genes

**Figure 5:**
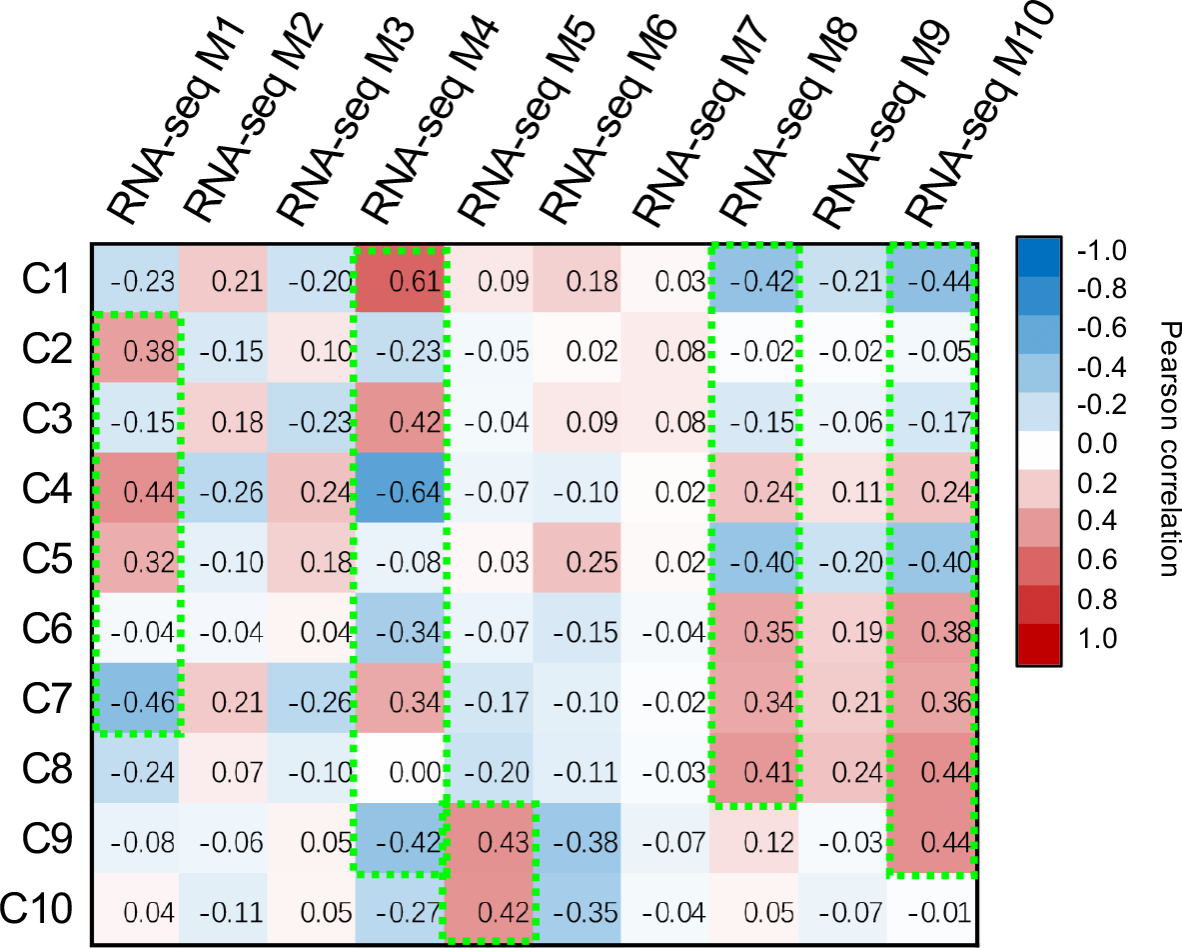
Pearson’s correlation analysis identifies the association between metabolic clusters and gene expression modules.

**Figure 6:**
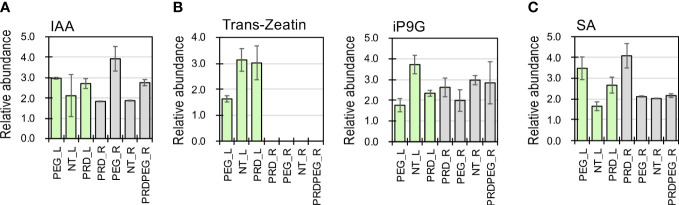
The relative abundance of several phytohormones (auxin, **(A)**; cytokinin, trans-Zeatin and iP9G, **(B)**; and salicylic acid, **(C)** detected using metabolomics.

To pinpoint the DEGs associated with the observed metabolic reprogramming under the PEG or PRD treatments, the gene co-expression modules were linked with the metabolic clusters using correlation analysis (**Figure 5**). Gene module M1 was positively correlated with metabolic clusters C2 and C4 but negatively correlated with C7. Gene module M4 was positively correlated with metabolic clusters C1 and C3 but negatively correlated with clusters C4 and C9. The module M5 was associated with the metabolic clusters C9 and C10. In addition, M8 and M10 were linked to the metabolic clusters C6 to C10. Among the identified DEMs, the abundance of several phytohormone compounds differed between the samples. For example, indole-3-acetic acid (IAA) was significantly higher in PEG_R than in PRD_R and NT_R (**Figure 6A**). Interestingly, the active form of cytokinin (trans-zeatin) was detected in the leaf tissues, with NT_L and PRD_L both having high levels of trans-zeatin but PEG_L having a low trans-zeatin content (**Figure 6B**). Additionally, a less active form of cytokinin (N6-isopentenyl adenine-9-glucoside, iP9G) was decreased in PEG_L and PRD_L when compared with NT_L (Hallmark and Rashotte, 2020; Chen et al., 2021). Cytokinin appears in several active forms (such as isopentenyl adenine (iP) or trans-zeatin(tZ)) and is involved in various processes related to cell division, leaf development and senescence, and abiotic stress resistance (Honig et al., 2018; Liu et al., 2020). More recently, a study has demonstrated that iP9G acts as a less active cytokinin compound and plays a role in delaying leaf senescence (Hallmark and Rashotte, 2020). The higher abundance of cytokinin metabolites in PRD_L could explain its physiological status similar to the non-treated leaves. Besides, in the root samples, NT_R had the lowest level of salicylic acid (SA), while PRD_R showed increased SA content. Similarly, in the leaf, NT_L exhibited low SA content, while PEG_R and PRD_R showed high levels of SA (**Figure 6C**), suggesting that SA signaling, and response might be involved in stress response and/or tolerance. Several metabolites associated with the metabolism of auxin, cytokinin, gibberellic acid, and ABA exhibited confound changes, implying complex regulation or coordination of the phytohormone metabolism and signaling during the response and adaptation to the osmotic stress (**Supplementary Figure S3**).

To substantiate the relationship between transcriptional regulation and metabolic reprogramming during osmotic stress tolerance, a transcription factor (TF)-centric approach was employed: (1) the transcription factor-encoding genes were annotated in the gene co-expression modules with particular focuses on those TFs in the modules highly correlated with the metabolic clusters (e.g., M1, M4, M5, M8, M9, and M10) (Jin et al., 2017); (2) By taking advantage of the numerous functional studies in rice, “TF- target genes – downstream affected metabolites” were mapped in our expression modules and metabolic clusters (Yao et al., 2018; Huang et al., 2022); (3) a collection of rice TFs with their regulated genes or potential target genes reported were used for enrichment analysis to reveal if any set of TF’s target genes is significantly enriched in a co-expression module in a genome-wide manner (details in the Method section “Integrated analysis of transcriptome and metabolome data”) (**Supplementary Table S7**).

**Figure 7:**
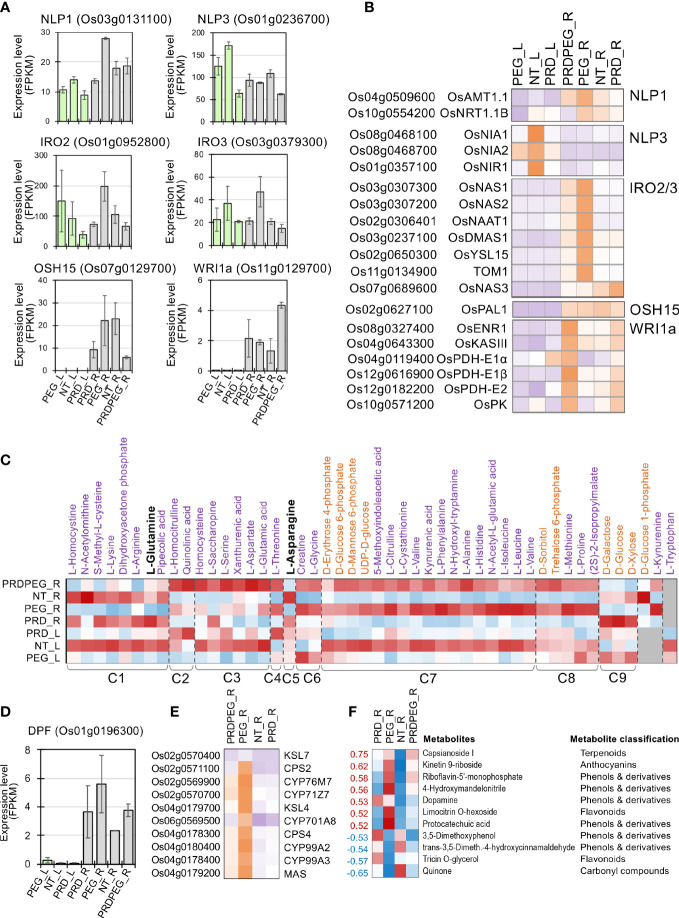
The metabolic reprogramming induced by PEG or PRD treatment and the underlying transcription factors. **(A)** Expression profiles of several key transcription factor genes (i.e., NLP1, NLP3, IRO2, IRO3, OSH15, and WRI1a) with validated functions in nitrogen utilization, ion transport and secondary metabolic pathways. **(B)** The expression profiles of the major target genes for NLP1, NLP3, IRO2, IRO3, OSH15, and WRI1a, respectively, in the PEG and PRD-treated samples. **(C)** Representative metabolites of amino acid and sugar metabolism showed dramatic changes, with PRD_R resembling the metabolic status of NT_R to maintain the supply of amino acids and sugars. Secondary metabolic pathways were affected **(F)** by either the PRD or PEG treatment partly through the differential expression of DPF **(D)**, a transcription factor controlling terpenoid biosynthesis, and its major target genes **(E)**.

Using these customized analysis approaches, we uncovered a complex coordination between primary metabolism (especially nitrogen transport and assimilation), ion transport and assimilation, and secondary metabolism in leaf and root tissues. First, two genes encoding NIN-like transcription factors (NIN-LIKE PROTEIN 1 and 3, NLP1 and NLP3, respectively) showed high expression levels in NT_L but lower expression in PEG_L and PRD_L (**Figure 7A**). PEG-treated roots also exhibited high OsNLP1 expression. Consistent with the upregulation of OsNLP1 in PEG_R, OsNLP1 is one of the major TFs involved in nitrogen utilization and transportation in rice and is primarily responsive to nitrogen deficiency (Alfatih et al., 2020). Indeed, the expression levels of OsNLP1’s primary target genes, the ammonium transporter OsAMT1.1 and the nitrate transporter OsNRT1.1B, were highly correlated with OsNLP1 (**Figure 7B**). OsAMT1.1 is a prominent member of the OsAMT family that controls ammonium uptake and assimilation and positively affects rice plants’ development and growth (Hoque et al., 2006; Ranathuge et al., 2014). In contrast, OsNRT1.1b is one of the major genes contributing to nitrate assimilation and divergence of NUE between japonica and indica rice subspecies, with several OsNRT1.1b natural variations being successfully used in rice breeding (Hu et al., 2015). In addition, OsNLP3 is highly expressed in the OsNLP family, with the highest expression levels detected in the green tissues (Zhang et al., 2022b). Correlated with OsNLP3 expression, the primary target genes of OsNLP3, encoding a set of nitrate reductase and nitrite reductase (i.e., OsNIA1, OsNIA2, and OsNIR1, respectively), were highly expressed in NT_L, but dramatically decreased in PEG_L and PRD_L (Tang et al., 2012b; Kabange et al., 2021), indicating that the nitrogen utilization ability in both PEG_L and PRD_L was probably compromised, while PRD_L leaves did not exhibit clear changes in several important physiological parameters (**Figure 1**).

In line with the expression patterns of the nitrogen-related TF OsNLP1 and its target genes observed in the root samples, it is found that most of the amino acids (AA) and AA derivatives exhibited similar abundances between NT_R and PRD_R, but in contrast to those in PRDPRG_R and PEG_R (**Figure 7C**). Nitrogen-rich AAs (glutamine and asparagine) are key to root-to-shoot nitrogen transportation, and their levels reflect nitrogen utilization and supply status (Hildebrandt et al., 2015; Galil et al., 2016). Importantly, both glutamine and asparagine were significantly abundant in NT_R and PRD_R but were lower in PRDPRG_R and PEG_R. By contrast, many amino acids and AA derivatives were present at low abundance in PEG_R and PRDPEG_R compared to those in NT_R/PRD_R, including branched-chain amino acids (valine, leucine, and isoleucine) and the S-containing AA methionine (**Figure 7C**). Together with the expression patterns of OsNLP1, OsNLP3, and their target genes in both the leaves and roots, these results indicate that nitrogen transport and assimilation are likely impaired in PEG and PRDPEG tissues, leading to systemic adjustments in multiple amino acid metabolic pathways.

Second, PEG-treated roots (PEG_R) showed significant upregulation of the Fe-deficiency-induced TFs IRO2 and IRO3 (ion-related transcription factors, IRO) (Ogo et al., 2011; Wang et al., 2021) (**Figure 7A**). Consistent with the expression patterns of OsIRO2 and OsIRO3, a group of key genes involved in Fe absorption and translocation was upregulated in PEG_R but not in the other root samples (Ogo et al., 2007). This pattern of OsIRO2/3 and its downstream target genes suggests that PEG-induced stress may lead to deficiencies in ion transportation and assimilation.

Third, changes in primary and secondary metabolic pathways were observed at both the transcriptome and metabolome levels. The *Oryza Sativa* Homeobox 15 gene (OSH15) encodes a class I KNOX protein involved in phenylpropanoid and lignin biosynthesis and organ development (Yoon et al., 2017). Indeed, the *PAL1* gene, which encodes the rate-limiting enzyme of phenylpropanoid biosynthesis, phenylalanine ammonia lyase 1 (PAL1) (Jun et al., 2018; Li et al., 2019b), shared a similar expression pattern with *OsOSH15*, which was highly expressed in NT_R and PEG_R, but lower in PRD_R and PRDPEG_R, implying repressed secondary metabolism to flavonoids and monolignol/lignin. In contrast, the *diterpenoid phytoalexin factor* (*DPF*) gene and its target genes related to the biosynthesis of diterpenoid phytoalexins (DPs) were highly expressed in PEG_R, moderately expressed in PRD_R and PRDPEG_R, and weakly expressed in NT_R (Yamamura et al., 2015) (**Figures 7D, E**). In addition, a group of phenol and terpenoid metabolites was up- or down-regulated, which correlated well with the changes observed at the transcriptional level (**Figure 7F**). The upregulation of terpenoid biosynthesis may be caused by PEG-induced stress but is more likely to reflect a reprogramming of secondary metabolites, in which terpenoid biosynthetic pathways related to pathogen defense are upregulated. Phenylpropanoid/lignin biosynthetic pathways are repressed to coordinate limited metabolic resources for stress response and adaptation.

In addition to stress-induced adjustments in nitrogen and secondary metabolism, osmolytes often accumulate in the root tissue in response to drought or osmotic stresses (Gorgues et al., 2022). Proline and sorbitol were highly accumulated in PEG_R and PRDPEG_R but remained at low levels in the NT_R and PRD_R samples (**Figure 7C**). In particular, a high level of proline was detected in PEG_L, indicating that proline is a major osmolyte in both leaves and roots that copes with PEG-induced osmotic stress. Another stress-related sugar metabolite, trehalose 6-phosphate, was abundant in PEG_R and PRDPEG_R. Unlike these stress-induced metabolites, sugars associated with primary metabolisms (especially glycolysis/gluconeogenesis), such as glucose, galactose, and glucose 1-phosphate, were low in PEG_R and PRDPEG_R but were low in NT_R and PRD_R (**Figure 7C**). This suggests that carbohydrate metabolism is likely to be affected by PEG treatment. This coordination between the metabolic pathways of carbohydrates, nitrogen/amino acids, and secondary compounds is at least partly controlled at the transcriptional level, with several functionally validated TFs (OsNLP1, OsNLP3, OsIRO2, OsIRO3, OsDPF, and OsOSH15) having clear roles in this coordination.

**Figure 8:**
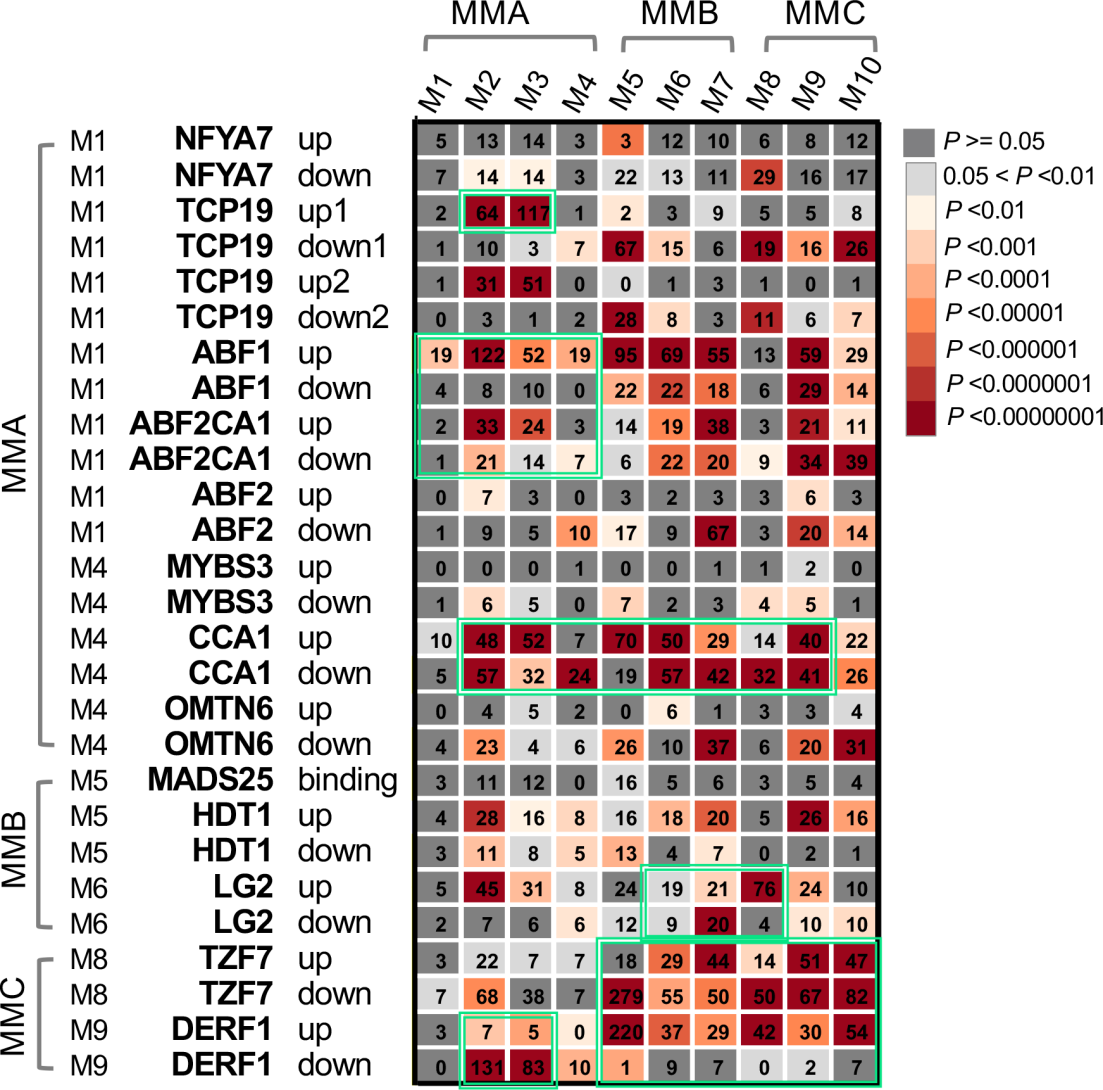
Significant overlaps between TF-target genes and the expression modules highlight several important transcriptional regulators in the modules. The heatmap shows the hypergeometric p values between TF-target genes and each of the ten expression modules, with dark grey indicating *P* < 0.05 and light grey indicating 0.05 < *P* < 0.01. Representative TFs in these modules and their potential regulated genes in each module are shown in the pie chart, with the representative MapMan functional terms listed for each set of TF-target genes. MMA, MMB, and MMC stand for the meta-module A, B, and C, respectively, which are provided in **Supplementary Figure S2**.

**Figure 9:**
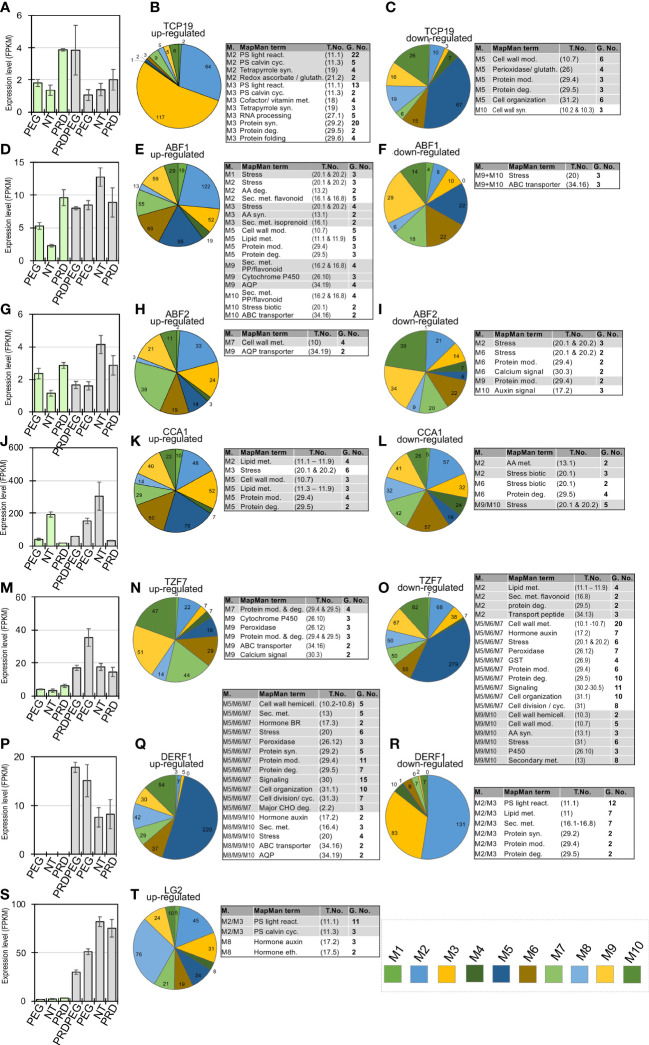
Profiling of the important transcription factors and regulators involved in the stress response and metabolic reprogramming during the PRD or PEG treatments. This diagram shows a total of seven selected transcription factors and regulators and their target genes or regulated genes in the coexpression modules (details in the Methods), with the expression profiles for TCP19, ABF1, ABF2, CCA1, TZF7, DERF1, LG2 shown in **(A, D, G, J, M, P, S)**, respectively. The up-regulated genes of TCP19, ABF1, ABF2, CCA1, TZF7, DERF1 and LG2 in each module are shown in pie charts in **(B, E, H, K, N, Q, T)**, respectively, with the genes annotated to be associated with stress and metabolic functional terms listed in the companion tables. The down-regulated genes of TCP19, ABF1, ABF2, CCA1, TZF7 and DERF1 in each module are shown in pie charts in **(C, F, I, L, O, R)**, respectively, with the genes annotated to be associated with stress and metabolic functional terms listed in the companion tables. In each table, the modules, MapMan-annotated term, term number (abbreviated as ‘T. No.’), and gene number (abbreviated as ‘G. No.’) of the TFregulated genes are provided.

To fully utilize the TF-centric analysis approach, functional studies of rice TFs were systematically searched, in which the TF-regulated or targeted genes were identified by comparing the TF transgenic line to the wild type using RNA-seq or chromatin immunoprecipitation sequencing (ChIP-seq) analysis (Su et al., 2010; Li et al., 2011; Wan et al., 2011; Tang et al., 2012a; Fang et al., 2014; Lee et al., 2015; Zhang et al., 2017; Xu et al., 2018; Zhang et al., 2018; Wang et al., 2019; Liu et al., 2021; Guo et al., 2022; Li et al., 2022; Wei et al., 2022). Several sets of the target genes for a total of 12 TFs were collected, including one stress-induced RNA-binding protein, tandem CCCH zinc finger 7, and TZF7 (**Supplementary Table S7**) and these gene sets were compared with the identified co-expression modules, with a significant overlap between a gene set and a module determined using a hypergeometric test (*P*< 0.01) (**Figure 8**) (Li et al., 2019a; Tu and Li, 2020). As expected, the TFs and their target genes tend to be significantly enriched within the same meta-modules (the modules with positively or negatively correlated co-expression patterns), highlighting a number of TFs involved in the transcriptional regulation, such as TCP19 (Teosinte branched1/Cincinnata/proliferating cell factor 19) (Liu et al., 2021), ABF1 (ABRE binding factor 1) (Zhang et al., 2017), ABF2 (ABRE binding factor 2) (Tang et al., 2012a), CCA1 (CIRCADIAN CLOCK ASSOCIATED 1), LG2 (LUGULELESS 2) (Li et al., 2022; Wei et al., 2022), TZF7 (Guo et al., 2022) and DERF1 (drought-responsive ethylene response factor 1) (Wan et al., 2011) (shown in green boxes in **Figure 8**). As osmotic or drought stress occurs, the key stress-responsive phytohormone ABA begins to accumulate, mainly in the leaf tissue, triggering its signal transduction and activating stress-related genes through several ABA-downstream TFs (Song et al., 2016; Chen et al., 2020). As expected, two major ABA-responsive TFs (i.e., ABF1 and ABF2) were highly expressed in PEG_L and PRD_L but not in NT_L (**Figures 9D, G**), and their upregulated genes were largely detected in modules M1 to M4 (**Figures 9E, H**). Interestingly, TCP19, which has been reported to be associated with rice nitrogen use efficiency (NUE) (Liu et al., 2021), was highly expressed in PRD-treated samples (PRD_L and PRDPEG_R). Given the distinct expression patterns of the other nitrogen-responsive genes (e.g., NLP1, NLP3, and their target genes) between NT-, PEG-treated-, and PRD-treated plants, and the similar metabolic changes in amino acid metabolism between NT_R and PRD_R, TCP19 might be a key regulator of nitrogen reprogramming in PRD-treated plants to coordinate root-to-shoot nitrogen allocation. Similarly, nitrogen reprogramming reflects the transcriptional regulation of multiple nitrogen transporters through several key TFs. Our analysis revealed that OsCCA1 (also known as N-mediated heading date 1, Nhd1) showed high expression exclusively in the untreated leaf and root tissues, which directly activated the ammonium transporter (OsAMT1;3) and the dual-affinity nitrate transporter (OsNRT2.4) to modulate nitrogen use efficiency (NUE) and root growth (Li et al., 2022; Wei et al., 2022). In contrast, the RNA-binding protein OsTZF7, belonging to the tandem CCCH zinc finger (TZF) family, was highly expressed in PEG-treated roots. OsTZF7 is an essential component of stress granules associated with the post-transcriptional regulation of mRNAs during stress and is responsive to drought stress and ABA signaling (Guo et al., 2022). Thus, TZF7 likely represents evidence of a PEG-induced ABA response that is distinct from the transcriptional regulation mediated by ABF1 and ABF2 in the leaf. We also detected similar expression patterns between NT_R and PRD_R for important transcription factors involved in stress tolerance and growth. OsDERF1 represents a novel ERF transcriptional cascade that modulates drought response through ethylene biosynthesis (Wan et al., 2011). Indeed, OsDERF1 remained at low expression levels in NT_R and PRD_R, with several hundred OsDERF1 target genes related to protein synthesis and cell wall metabolism that were well correlated in the MMC meta-module (**Figures 8A**, **9P–R**). LIGULELESS2 (LG2) is another key transcription factor involved in leaf organogenesis; however, its role in root development remains elusive (Wang et al., 2021). The upregulated expression of LG2 in NT_R and PRD_R, together with its regulated genes, suggests that LG2 may play a role in maintaining root growth.

Besides, the differentially expressed genes identified in this work were compared with the rice meta-quantitative trait loci (MQTLs) that are known to involved in drought-related traits (Khahani et al., 2021). In particular, several MQTL regions related to root architecture-related traits were focused, including RDR (ratio of deep rooting), RDW (root dry weight), RL (root length), RN (root number), and RT (root thickness). 745 DEGs in our study were found to be co-localized with the MQTLs (**Supplementary Tables S9, S10**). Interestingly, the four drought-tolerance MQTL regions do not enrich the DEGs, whereas four, two four, and one MQTL regions associated with RT, RN, RDW and RDR traits are significantly enriched with the DEGs related to PRD-mediated stress tolerance. Several stress-related regulators identified in the present study were found in these MQTL regions, such as ABF1, TZF7, NF-YA7, and OMTN6 (**Supplementary Table S9**). Thus, the DEGs co-localized with drought related MQTLs may represent a useful source of candidate genes associated with drought tolerance in rice. Combining the results of TF-centric analysis and MQTL analysis, several regulators involved in stress response and metabolic changes (ABF1, ABF2, CCA1, DERF1, IRO2/3, LG2, NLP1, TCP19, TZF7, WRI1a) could be prioritized as major candidate genes for identifying rice genotypes with better PRD-mediated stress tolerance, while the DEGs co-localized with the drought related MQTL regions also deserving future investigations.

## Conclusion

**Figure 10:**
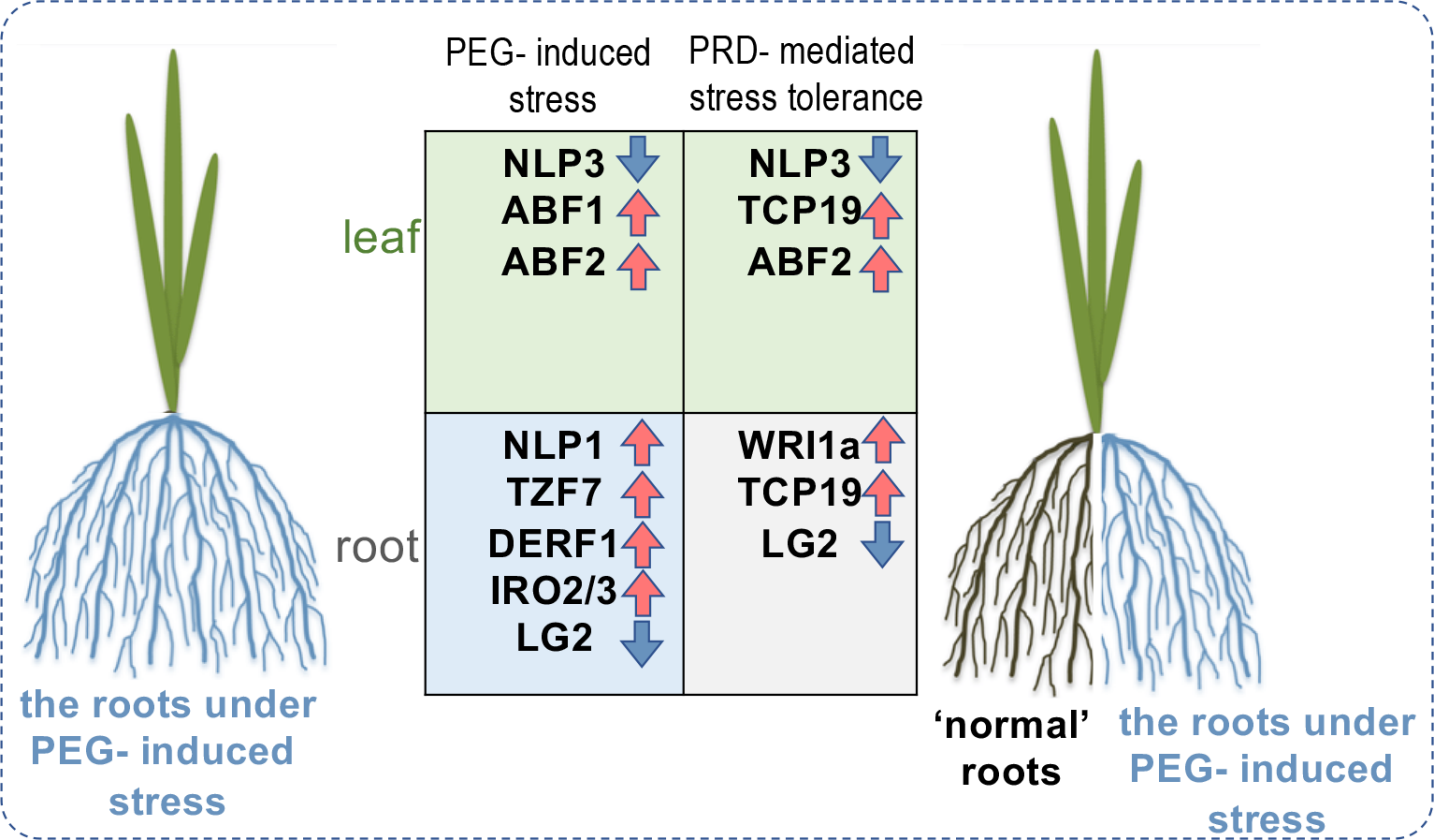
Proposed model illustrating the molecular differences between PEG-stressed and PRD-mediated stress-tolerant rice seedlings, highlighting a couple of potentially involved key regulators including not only the transcription factors (*e.g.*, ABF1, ABF2) downstream of ABA signaling pathway, but also several regulators related to metabolism and nutrient uptake (*e.g.*, NLP3, TCP19, WRI1a).

In this study, we integrated the results from physiological, transcriptome, and metabolome analyses. Our results demonstrated that PRD induces transcriptomic changes primarily in the roots but not in the leaves and adjusts several amino-acid and phytohormone metabolic pathways to maintain the balance between growth and stress responses. To the best of our knowledge, this is the first report to employ an integrated omics analysis to decipher the molecular regulation underlying the PRD technique. Our results established a link between transcriptional regulation and PRD-induced metabolic reprogramming. More importantly, transcription factors were identified in these co-expression modules, highlighting several key TFs, such as TCP19, WRI1a, ABF1, ABF2, DERF1, and TZF7, involved in nitrogen metabolism, lipid metabolism, ABA signaling, ethylene signaling, and stress regulation (summarized in **Figure 10**). These identified regulators and the associated transcriptional and metabolic changes strongly support the involvement of other mechanisms (such as metabolic reprogramming) in PRD-mediated stress tolerance. It is also worth mentioning that the main aim was to provide evidence for the hypothesis mentioned in the Introduction. However, more molecular experiments are necessary to validate the detailed functions of each regulator in the PRD process. Overall, our results provide new insights into PRD-mediated osmotic stress tolerance, clarify the molecular regulation induced by PRD, and identify genes useful for further improving water-use efficiency and/or stress tolerance in rice.

## Data availability statement

The original contributions presented in the study are publicly available. This data can be found here: NCBI Short Read Archive (SRA), accession number PRJNA936260 (https://www.ncbi.nlm.nih.gov/sra/?term=PRJNA936260).

## Author contributions

Conceptualization, JZ, YL, and XC. Experiments and methodology, MZ, CD, JZ, ZG, YYZ, JW, YPZ, YL, and YaW. Data acquisition and analyses, YaW, MC, YuW, JC, GY, and GH. Bioinformatic analyses, MZ, CD, JZ, and YL. Writing—original draft preparation, MZ, CD, YL, and XC. Writing—review and editing, MZ, CD, YL, and XC. Supervision, YL, and XC. Project administration, JZ, JC, GY, GH, YL, and XC. Funding acquisition, JZ, YL, and XC. All authors contributed to the article and approved the submitted version.
