# Supplementary material for: Integrated omic analysis provides insights into the molecular regulation of stress tolerance by partial root-zone drying in rice
Source: Front Plant Sci. 2023 Jun 9;14:1156514. doi: 10.3389/fpls.2023.1156514 (PMC10288491; doi:10.3389/fpls.2023.1156514)
Supplement: Supplementary file 1 [file DataSheet_1.zip › Figures S1-S3 and Tables S7-S9.docx]

**Supplementary Information for**

Integrated Omic Analysis Provides Insights into the Molecular Regulation of Stress Tolerance by Partial Root-zone Drying in Rice

**Minhua Zhao ^1,#^, Canghao Du ^2,#^, Jian Zeng ^1^, Zhihong Gao ^1^, Yongyong Zhu ^1^, Jinfei Wang ^1^, Yupeng Zhang ^1^, Zetao Zhu ^1^, Yaqiong Wang ^2^, Mingjie Chen ^2^, Yuesheng Wang ^2^, Junli Chang ^2^, Guangxiao Yang ^2^, Guangyuan He ^2,*^, Yin Li ^2,*^, and Xiaoyuan Chen ^1,*^**

1 Henry Fok School of Biology and Agriculture, Guangdong Engineering Technology Research Center for Efficient Utilization of Water and Soil Resources in North Region, Shaoguan University, Shaoguan, Guangdong 512005, China;

2 The Genetic Engineering International Cooperation Base of Chinese Ministry of Science and Technology, Key Laboratory of Molecular Biophysics of Chinese Ministry of Education, College of Life Science and Technology, Huazhong University of Science and Technology, Wuhan, Hubei, China;

* Correspondence: hegy@hust.edu.cn; chenxy2@163.com; yinli2021@hust.edu.cn;

# These authors contributed equally to this work.


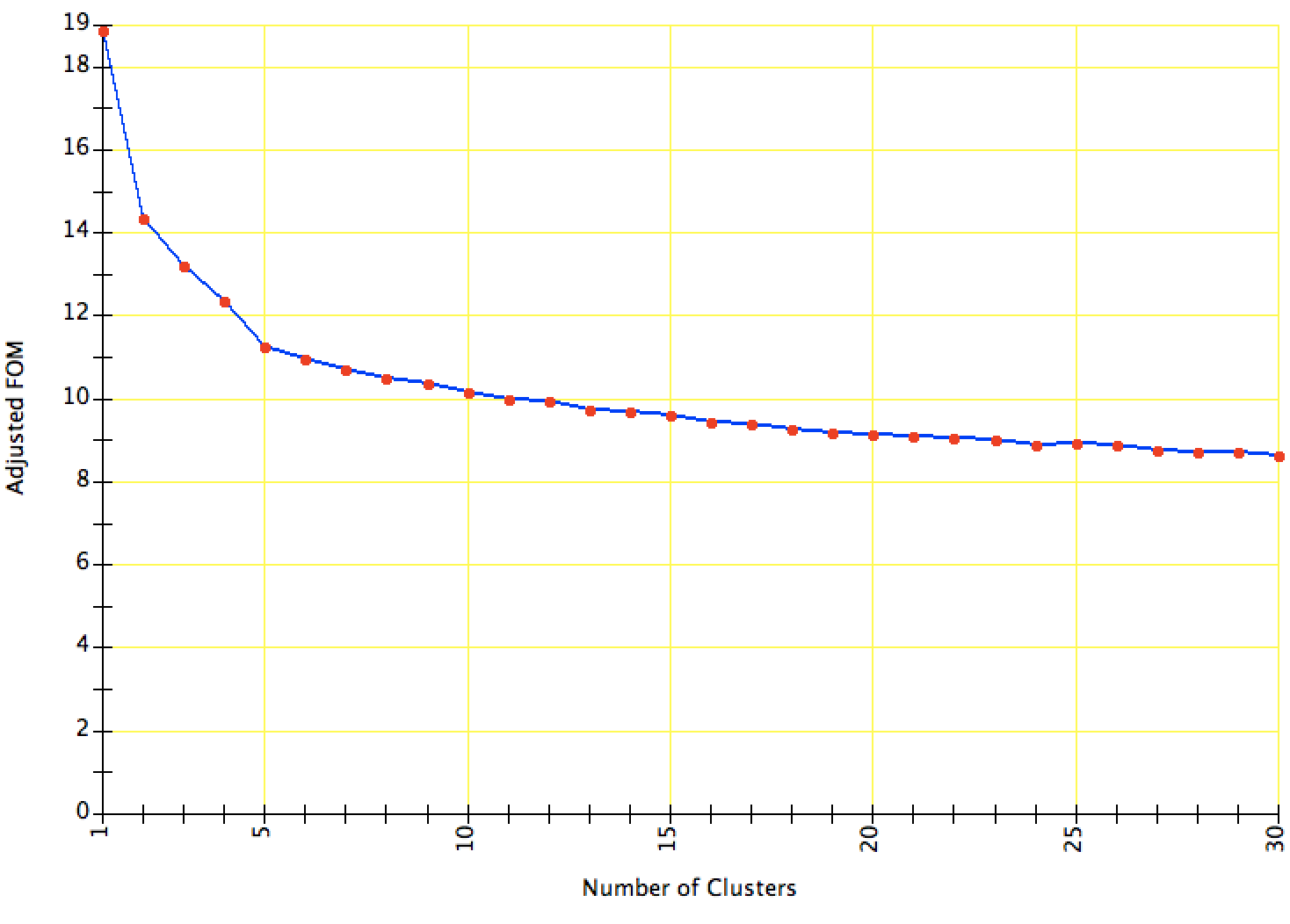


**Figure S1** | Figure of merit (FOM) analysis result for the transcriptomic k-means clustering.


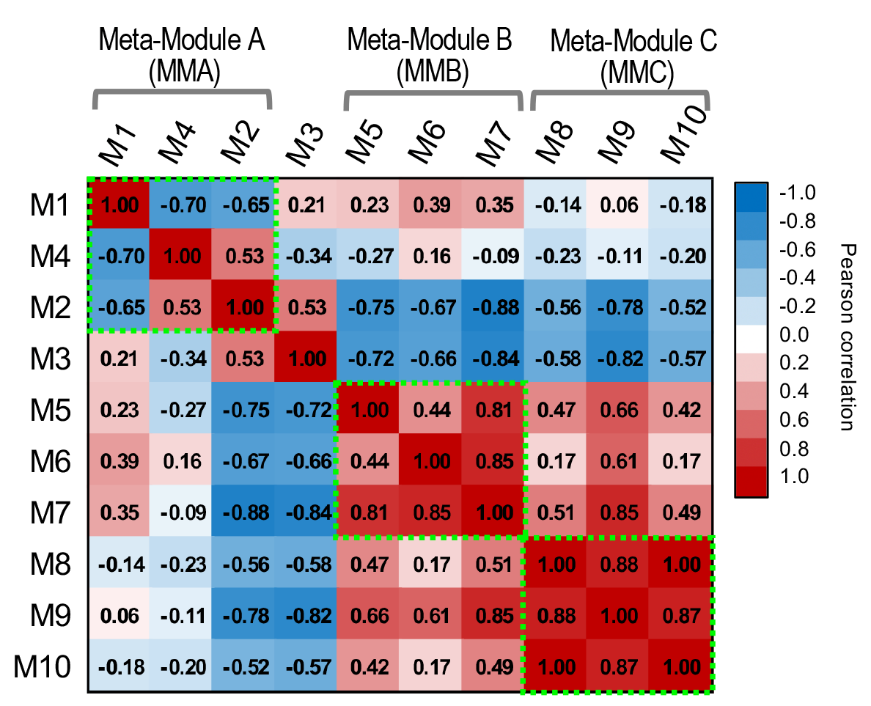


**Figure S2** | Correlation analysis between the representative expression patterns for each module reveals that the gene modules could be aggregated into three major meta-modules, Meta-modules A, B and C (abbreviated as MMA, MMB and MMC, respectively).


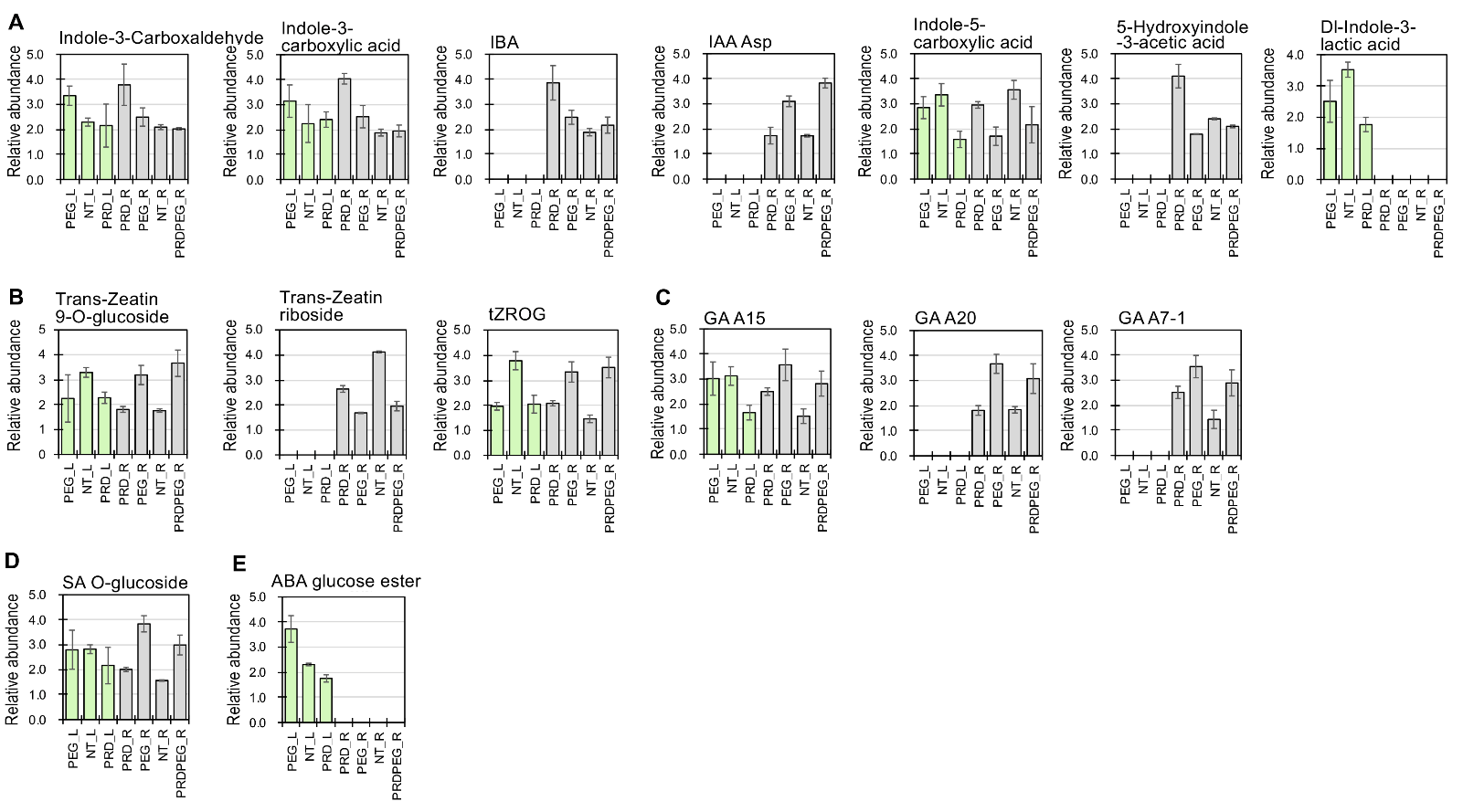


**Figure S3 |** Relative abundance of the metabolites involved in several phytohormone metabolic pathways, including auxin **(A)**, cytokinin **(B)**, gibberellic acid **(C)**, salicylic acid **(D)** and abscisic acid **(E)**.

**Table S7** | A summary of the TFs and regulators used for the TF-centric analysis.

| **TF/**  **regulators** | **geneID** | **Type** | **References** | **Major target genes** | **Note** |
| --- | --- | --- | --- | --- | --- |
| NLP1 | Os03g0131100 | 1 | Alfatih et al. 2020 *J. Exp. Botany* | OsAMT1.1, OsNRT1.1B | Not applicable |
| NLP3 | Os01g0236700 | 1 | Zhang et al. 2022 *Plant Cell Environ.* | OsNIA1, OsNIA2, OsNir1 | Not applicable |
| IRO2 | Os01g0952800 | 1 | Ogo et al. 2011 *Plant Mo. Biol.* | OsNAS1, OsNAS2, OsNAAT1, OsDMAS1, OsYSL15, TOM1 | Not applicable |
| IRO3 | Os03g0379300 | 1 | Wang et al. 2020 *Plants* | OsNAS1, OsNAS2, OsNAS3 | Not applicable |
| OSH15 | Os07g0129700 | 1 | Yoon et al. 2017 *Plant Physiol.* | OsPAL1 | Not applicable |
| WRI1a | Os11g0129700 | 1 | Liu et al. 2022 *PLoS One* | OsENR1, OsKAS III, OsPDH-E1α, OsPDH-E1β, OsPDH-E2, OsPK | Not applicable |
| DPF | Os01g0196300 | 1 | Yamamura et al. 2015 *Plant J.* | OsKSL7, OsCPS2, OsCYP76M7, OsCYP71Z7, OsKSL4, OsCYP701A8, OsCPS4, OsCYP99A2, OsCYP99A3, OsMAS | Not applicable |
| TCP19 | Os06g0226700 | 2 | Liu et al. 2020 *Nature*  Mukhopadhyay et al. 2015 *Sci. Rep.* | Not applicable | TCP19 is responsive to nitrogen availablity and functions in nitrogen use efficiency and root development. |
| ABF1 | Os01g0867300 | 2 | Zhang et al. 2017 *J. Exp. Botany* | Not applicable | ABF1 (also known as ABI5) is one of the key TFs responsive to ABA in rice and involved in ABA-dependent drougtht resistance throught directly regulating several hundreds of target genes regarding multiple aspects of the drought response. |
| ABF2 | Os06g0211200 | 2 | Tang et al. 2012 *Plant Physiol.* | Not applicable | ABF2 is one of the key TFs in ABA signaling pathway in rice and a positive regulator of drought stress tolerance. |
| CCA1 | Os08g0157600 | 2 | Wei et al. 2022 *Plant Physiol.*  Li et al. 2022 *Plant Physiol.* | Not applicable | CCA1 is a vital component of the circadian clock in rice, which can regulate several hundreds of genes, including many core components of the ABA signaling, such as PP2C and OsbZIP46. |
| TZF7 | Os05g0525900 | 2 | Guo et al. 2022 *Front. Plant Sci.* | Not applicable | TZF7 encodes an RNA binding protein responsive to ABA and abiotic stresses, is associated with stress granules and can recognized the adenine and uridine-rich element (ARE) and ARE-like motif of the mRNA. |
| DERF1 | Os08g0454000 | 2 | Wan et al. 2011 *PLoS One* | Not applicable | DERF1 encodes an ERF TF protein responsive to drought, ethylene and ABA and functions as a negative regulator of drought resistance. |
| LG2 | Os01g0859500 | 2 | Wang et al. 2020 *New Phytologist* | Not applicable | LG2 encodes a bZIP TF protein known to be essential for the lamina joint (LJ) organogenesis and leaf angle. |
| NF-YA7 | Os08g0196700 | 2 | Lee et al. 2015 *Plant Sci.* | Not applicable | NF-YA7 is a positive regulator of drought resistance to improve drought tolerance through an ABA-independent manner. |
| MYBS3 | Os10g0561400 | 2 | Su et al. 2010 *Plant Physiol.* | Not applicable | MYBS3 positively regulates cold stress in rice and mediates sugar signaling, while it also represses the key regulator of cold response DREB1/CBF. |
| OMTN6 | Os08g0200600 | 2 | Fang et al. 2014 *J. Exp. Botany* | Not applicable | OMTN6 is a typical NAC TF protein regulated by miR164 and its overexpression negatively affects drought resistance. |
| MADS25 | Os04g0304400 | 2 | Zhang et al. 2018 *Plant J.*  Xu et al. 2018 *PLoS Genet.* | Not applicable | MADS25 is a positive regulator of oxidative stress in rice and regulates ROS scavenging activity in rice roots. Overexpression of MADS25 can enhance salinity and oxidative stress tolerance. |
| HDT1 | Os05g0597100 | 2 | Li et al. 2011 *PLoS One* | Not applicable | While mis-annotated as a TF, HDT1 is acutally a histone deacetylase regulating several hundreds of genes’ expression through epigenetics, for which overexpression leads to early flowering. |

Note: 1. Major target genes or regulated genes have been functionally validated; 2. The target genes or regulated genes have been characterized by genome-wide expression techniques (i.e., microarray or RNA-seq) for these TFs or regulators.

**Table S8** | RNA-seq statistics for this study.

| **Sample_name** | **Raw reads** | **Clean reads** | **Q20_rate** | **Q30_rate** | **Total_read pair (bp)** | **Percent of the unique_mapped reads (%)** |
| --- | --- | --- | --- | --- | --- | --- |
| NT_L_1 | 43,382,154 | 43,341,084 | 98.70% | 95.80% | 21,670,542 | 93.78 |
| NT_L_2 | 43,339,748 | 43,301,242 | 98.60% | 95.50% | 21,650,621 | 90.79 |
| NT_L_3 | 43,337,788 | 43,293,124 | 98.80% | 96.00% | 21,646,562 | 92.62 |
| PRD_L_1 | 43,365,910 | 43,331,728 | 98.70% | 95.90% | 21,665,864 | 91.39 |
| PRD_L_2 | 43,364,876 | 43,321,038 | 98.90% | 96.40% | 21,660,519 | 77.62 |
| PRD_L_3 | 43,345,888 | 43,317,212 | 99.00% | 96.50% | 21,658,606 | 91.65 |
| PEG_L_1 | 43,361,776 | 43,333,898 | 98.50% | 95.00% | 21,666,949 | 89.67 |
| PEG_L_2 | 43,383,208 | 43,344,096 | 98.70% | 95.90% | 21,672,048 | 92.31 |
| PEG_L_3 | 32,212,372 | 32,193,294 | 98.80% | 96.10% | 16,096,647 | 91.43 |
| NT_R_1 | 43,345,294 | 43,308,920 | 98.80% | 96.00% | 21,654,460 | 85.77 |
| NT_R_2 | 43,398,700 | 43,356,388 | 99.00% | 96.60% | 21,678,194 | 85.6 |
| NT_R_3 | 43,391,168 | 43,355,268 | 99.10% | 97.00% | 21,677,634 | 76.53 |
| PRD_R_1 | 43,391,474 | 43,356,684 | 98.90% | 96.40% | 21,678,342 | 61.73 |
| PRD_R_2 | 43,394,334 | 43,358,228 | 98.90% | 96.10% | 21,679,114 | 76.84 |
| PRD_R_3 | 43,369,428 | 43,338,056 | 98.70% | 95.80% | 21,669,028 | 64.33 |
| PRDPEG_R_1 | 43,369,818 | 43,335,098 | 98.80% | 96.00% | 21,667,549 | 64.43 |
| PRDPEG_R_2 | 43,357,116 | 43,323,992 | 98.70% | 95.80% | 21,661,996 | 72.47 |
| PEG_R_1 | 43,378,232 | 43,336,534 | 98.80% | 96.00% | 21,668,267 | 44.84 |
| PEG_R_2 | 43,348,568 | 43,294,320 | 99.00% | 96.50% | 21,647,160 | 70.11 |
| PEG_R_3 | 43,363,776 | 43,326,732 | 98.70% | 95.80% | 21,663,366 | 62.65 |

**Table S9** | Overlapping between rice drought-related QTL regions and the identified DEGs. DT, drought tolerance; RT, root thickness; RN, root number; RL, root length; RDR, ratio of deep rooting; RDW, root dry weight.

| **Trait** | **Chr** | **Flanking markers** | **QTL position on the consensus reference map (cM)** | **Expressed genes**  **in the QTL** | **DEGs**  **in the QTL** | **P values** | **Co-localized**  **regulators** |
| --- | --- | --- | --- | --- | --- | --- | --- |
| DT | 1 | RM7318-C10728S | 113.16 | 51 | 12 | 0.1264 |  |
| DT | 4 | C12216S-E61747S | 41.52 | 4 | 1 | 0.4219 |  |
| DT | 8 | RM7356-S11134 | 93.44 | 18 | 4 | 0.2124 |  |
| DT | 11 | E20817-E3558S | 74 | 45 | 12 | 0.1298 |  |
| RT | 1 | C409-RM7566 | 111.11 | 210 | 57 | ***0.0494*** |  |
| RT | 1 | E50125S-RM5759 | 150.68 | 208 | 73 | ***0.0003*** | ABF1 |
| RT | 4 | C1087-C377 | 70.17 | 211 | 54 | 0.0623 |  |
| RT | 5 | RM3381-RM5948 | 62.56 | 358 | 91 | ***0.0483*** |  |
| RT | 6 | RM8112-RM584 | 13.32 | 149 | 32 | ***0.0462*** |  |
| RT | 9 | RM3787-C482 | 123.47 | 115 | 29 | 0.0856 |  |
| RN | 1 | RM2772-C808 | 104.4 | 128 | 43 | ***0.0074*** |  |
| RN | 5 | RM5401-RM2457 | 100.8 | 421 | 113 | ***0.0316*** | TZF7 |
| RN | 9 | RM3808-C482 | 124.14 | 62 | 15 | 0.1161 |  |
| RL | 4 | RM6992-RM6909 | 105.97 | 149 | 38 | 0.0746 |  |
| RL | 9 | C2985-C397 | 81.42 | 27 | 8 | 0.1443 |  |
| RL | 9 | S4677S-RM6839 | 92.16 | 53 | 15 | 0.1052 |  |
| RL | 9 | C12357S-RM6643 | 132.34 | 20 | 5 | 0.2024 |  |
| RDR | 2 | R418-RM6424 | 124.07 | 66 | 16 | 0.1126 |  |
| RDR | 4 | RM5320-R2737 | 91.65 | 87 | 30 | ***0.0134*** |  |
| RDR | 9 | RM5526-RM7038 | 78.11 | 125 | 28 | 0.0665 |  |
| RDW | 1 | E50125S-RM6593 | 148.37 | 97 | 37 | ***0.0015*** |  |
| RDW | 5 | E417S-RM3631 | 104.43 | 158 | 44 | 0.052 |  |
| RDW | 8 | RM8266-RM8256 | 53.06 | 177 | 60 | ***0.0021*** | NF-YA7; OMTN6 |
| RDW | 8 | S11102-RM8043 | 103.72 | 43 | 19 | ***0.003*** |  |
| RDW | 9 | RM3909-C11503S | 120.57 | 37 | 12 | 0.0844 |  |
| RDW | 11 | S2137-C61883S | 57.03 | 48 | 15 | 0.078 |  |
| RDW | 11 | RM7240-RM6688 | 119.95 | 23 | 12 | ***0.0035*** |  |
